# Microstructure and Properties of TiB_2_ Composites Produced by Spark Plasma Sintering with the Addition of Ti_5_Si_3_

**DOI:** 10.3390/ma14143812

**Published:** 2021-07-08

**Authors:** Agnieszka Twardowska, Marcin Podsiadło, Iwona Sulima, Krzysztof Bryła, Paweł Hyjek

**Affiliations:** 1 Institute of Technology, Pedagogical University, 2 Podchorazych, 30-084 Krakow, Poland; iwona.sulima@up.krakow.pl (I.S.); krzysztof.bryla@up.krakow.pl (K.B.); pawel.hyjek@up.krakow.pl (P.H.); 2 Łukasiewicz Research Network–Krakow Institute of Technology, 73 Zakopianska, 30-418 Krakow, Poland; marcin.podsiadlo@kit.lukasiewicz.gov.pl

**Keywords:** TiB_2_, Ti_5_Si_3_, spark plasma sintering, hardness, friction–wear properties

## Abstract

Titanium diboride (TiB_2_) is a hard, refractory material, attractive for a number of applications, including wear-resistant machine parts and tools, but it is difficult to densify. The spark plasma sintering (SPS) method allows producing TiB_2_-based composites of high density with different sintering aids, among them titanium silicides. In this paper, Ti_5_Si_3_ is used as a sintering aid for the sintering of TiB_2_/10 wt % Ti_5_Si_3_ and TiB_2_/20 wt % Ti_5_Si_3_ composites at 1600 °C and 1700 °C for 10 min. The phase composition of the initial powders and produced composites was analyzed by the X-ray diffraction method using CuK_α_ radiation. The microstructure was examined using scanning electron microscopy, accompanied by energy-dispersive spectroscopy (EDS). The hardness was determined using a diamond indenter of Vickers geometry loaded at 9.81 N. Friction–wear properties were tested in the dry sliding test in a ball-on-disc configuration, using WC as a counterpart material. The major phases present in the TiB_2_/Ti_5_Si_3_ composites were TiB_2_ and Ti_5_Si_3._ Traces of TiC were also identified. The hardness of the TiB_2_/Ti_5_Si_3_ composites was in the range of 1860–2056 HV1 and decreased with Ti_5_Si_3_ content, as well as the specific wear rate W_v_. The coefficient of friction for the composites was in the range of 0.5–0.54, almost the same as for TiB_2_ sinters. The main mechanism of wear was abrasive.

## 1. Introduction

The most extensively studied phase in the Ti-B system is titanium diboride, as it is the hardest and most thermodynamically stable. It is characterized by an extremely high melting point (3225 °C), high hardness (35–40 GPa) and Young’s modulus (450 GPa), which are associated with relatively high thermal (60–120 W/mK) and electrical conductivities (~10^5^ S/cm) [1]. Due to this unique set of properties, titanium diboride is an attractive material for a number of applications, including cutting tools, wear-resistant parts and coatings, ballistic armor, cathodes in Hall–Heroult cells for aluminum smelting, crucibles for handling molten metals and metal evaporation boats [1,2]. TiB_2_ crystallizes in a hexagonal closely packed crystal lattice (P6/mmm space group) with elementary unit cell parameters of a = 3.02 Å and c = 3.22 Å. The anisotropy of its thermal expansion coefficient is believed to be a source of microcrack formation and brittleness, observed either at the synthesis stage of TiB_2_ or thermal cycles taking place afterward both in bulk materials and thin films [3]. A low self-diffusion coefficient and an extremely high melting point make it difficult to obtain TiB_2_ sinters of high density [4,5]. Among the different sintering methods, the most effective and fastest is spark plasma sintering (SPS). It was demonstrated that by the SPS method, using simultaneously pulsed direct current and uniaxial pressure, TiB_2_ sinters of 96% theoretical density could be produced within 10 min at a temperature range of 1200–1800 °C, but the produced sinters were susceptible to cracking. To limit the brittleness of TiB_2_ sinters, sintering aids have been introduced. The use of sintering additives is advantageous for the density and temperature of sintering. Metal additives are effective in lowering the sintering temperature, but at the cost of lowering the mechanical properties. Among the ceramic aids used for TiB_2_ sintering are TiC [6], MoSi_2_ [7] and TiSi_2_ [8,9]. It was demonstrated that TiB_2_/TiSi_2_ composites with a TiSi_2_ content of 5–15 wt % produced by SPS at a temperature range of 1200–1400 °C can achieve high density (~98% of the theoretical density) [9]. It was found that the addition of TiSi_2_ enhances the densification, lowers the temperature of sintering and is beneficial for the mechanical properties of TiB_2_-based composites; however, due to the melting point of TiSi_2_, which is 1464 °C, the working temperature of the produced composites is limited. There are four titanium silicides in the Ti-Si system, among them Ti_5_Si_3_ (Mn_5_Si_3_ crystal structure type), which is the most thermodynamically stable [10]. In comparison to TiSi_2_, Ti_5_Si_3_ is characterized by a lower density (4.32 g/cm^3^), a higher melting point (2130 °C), higher hardness and resistance to oxidation, so it is also an attractive material to be used as a sintering aid for TiB_2_-based composites [8]. Research on the use of this titanium silicide as a sintering additive is scarce because of its brittleness, which may adversely affect the properties of the obtained composites. Therefore, most studies concern the content of this silicide up to 5%. The aim of this study is to densify TiB_2_/Ti_5_Si_3_ micropowders by the SPS method and to study the influence of Ti_5_Si_3_ addition on the microstructure, hardness and friction–wear properties of the produced composites.

## 2. Materials and Methods

### 2.1. Powders, Powder Mixtures and Parameters of Their Homogenization

Two commercially available powders were used: TiB_2_ (Sigma-Aldrich, Darmstadt Germany) of 99.9 wt % chemical purity, grain size = <10 µm, density = 4.52 g/cm^3^; Ti_5_Si_3_ (Alfa Aesar, Kandel, Germany of 99.5 wt % chemical purity, grain size = <10 µm, density = 4.32 g/cm^3^. Two powder mixtures were investigated: TiB_2_ + 10 wt % Ti_5_Si_3_ and TiB_2_ + 20 wt. % Ti_5_Si_3_. The powder mixtures were homogenized three times in SpeedMixer TM DAC 400.1 FVZ (Hauschild & Co. KG, Hamm, Germany) at a rotational speed of 2000 RPM and a rotation time of 30 s, followed by a 10 s break.

### 2.2. Spark Plasma Sintering

Spark plasma sintering was conducted using FCT Systeme GmbH (Effelder-Rauenstein, Germany), model HP D 5, and graphite die with an inner diameter of 20 mm. Densification of powders started with initial degassing by pressing for 10 min in vacuum (5 × 10^−1^ mbar) to the maximum pressure of 35 MPa. After that, the furnace chamber was filled with argon, heated up at a rate of 200 °C/min to the selected sintering temperature (1600 °C and 1700 °C), held for 10 min then cooled down at a rate of 200 °C/min.

### 2.3. Relative Density, Microstructure Examination

Sintered samples were mechanically ground and polished on one side using diamond grinding (9, 6 and 3 μm) and polishing suspensions (1 μm), respectively. At each grinding step, samples were degreased in isopropanol, ultrasonically cleaned in distilled water for 5 min and dried in air. The relative densities of the composites were determined by the Archimedes immersion method by measuring the weight differences of the specimens in air and in ethanol pure at room-temperature conditions. The theoretical densities of the samples were calculated according to the rule of mixtures. Phase compositions of powders and sinters were analyzed by the X-ray diffraction method (XRD) in Bragg–Brentano geometry using CuKα radiation (*λ* = 1.5406 Å, *U* = 40 kV, I = 30 mA). The XRD patterns were collected in 2θ geometry over scattering angles ranging from 20° to 90° with a step size of 0.02°. Phase identification was performed according to The International Centre for Diffraction Data (ICDD^®^) database. The microstructure of the samples was observed by the scanning electron microscope (SEM) JEOL JSM-6610 LV (JEOL Ltd., Tokyo, Japan) with energy-dispersive X-ray spectroscopy (EDS) using the X-Max detector (Oxford Instruments, Abingdon, UK), equipped with Aztec 2.1 software.

### 2.4. Hardness and Friction–Wear Tests

**Figure 1:**
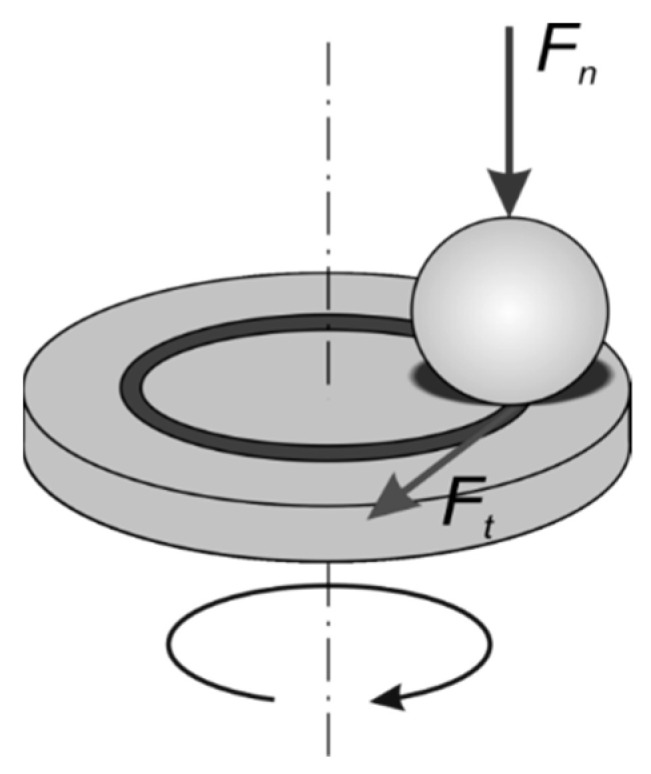
Ball-on-disc geometry of the friction–wear test.

Hardness was determined using the NEXUS 4000 hardness tester (Innovatest Europe bv, Maasstricht, The Netherlands) by the Vickers method using a diamond indenter loaded at 9.81 N. Five indents were made in three different areas of the polished surface. The friction–wear properties of the composites and referential TiB_2_ sinter sample of 97% relative density were tested in the dry sliding test in a ball-on-disc geometry (Figure 1), according to the ASTM G99-95a standard (reapproved in 2000) [11]. As a counterpart material, WC (6 wt % Co) was used. Balls 3.175 mm in diameter were loaded at 5 N. The sliding velocity was 0.1 m/s, while the total sliding distance was 200 m. The test duration was 2000 s, and the diameter of the wear track was 10 mm. Friction–wear tests were conducted in air at a relative humidity of 40–45% at room-temperature conditions. The friction force F_t_ was measured continuously during the test using an extensometer. For each test, a new WC ball was used. Before each test, both the ball and the sample were washed in high-purity acetone, dried in air and weighted. After mounting in the holder, the ball and the sample were washed again (in ethanol) then dried. The coefficient of friction (CoF) were calculated according to Equation (1):

CoF = F_t_/F_n_ (1)

where F_t_ is the measured friction force (N), and F_n_ is the load applied (N).

The specific wear rate of the specimens (disc and ball) was calculated from the mass difference of each specimen before and after the ball-on-disc tests, according to Equation (2):

(2) $$W_{v}=\frac{m_{a}\;-{\;m}_{b}}{\mathrm{Fn}\;\times \;d\;\times \;L}$$

where W_v_ is the specific wear rate (mm^3^/Nm), m_a_ is the mass before the test (kg), m_b_ is the mass after the test (kg), F_n_ is the applied load (N) and d is the theoretical density (kg/m^3^).

The microstructure of the composites was observed using SEM before and after the friction–wear test. Microstructure observations were accompanied by EDS analysis.

## 3. Results

### 3.1. SPS Sintering

**Figure 2:**
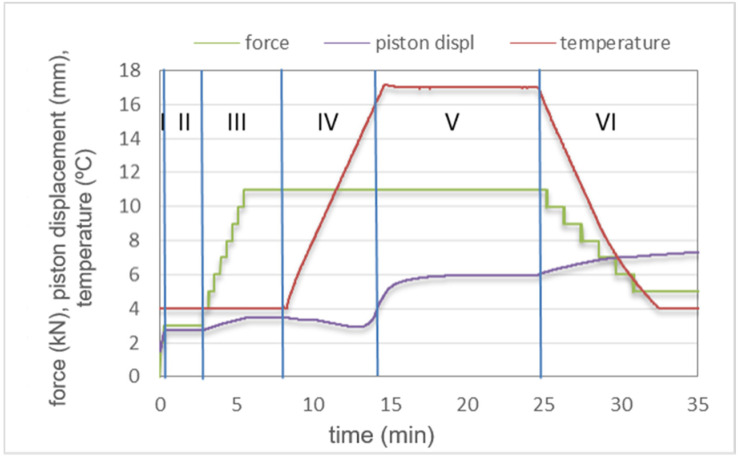
Experimental plot showing changes in applied force, piston displacement and temperature during the SPS sintering of the TiB_2_/20 wt % Ti_5_Si_3_ powders (t_max_ 1700 °C/10 min).

The variation of temperature, applied force (pressure), punch displacement (piston movement) and sintering speed during the SPS cycle is shown in Figure 2. The observed changes in the piston displacement determine the five stages of changes that take place during sintering (I–VI) to represent the accompanying phenomena. In Stage I, particle rearrangement takes place initiated by the applied force. A positive punch displacement is recorded (Stages I and III). In between (Stage II), degassing of the powder mixture under constant temperature and force takes place. In Stage IV, a slight negative piston displacement is recorded, caused by a temperature increase from 400 °C up to the requisite sintering temperature of 1700 °C. At Stage V, the piston, after initial positive displacement, stabilizes at a constant level. At this stage, the pores have to be eliminated. The last stage (Stage VI) is the cooling stage. Further positive piston displacement is observed due to thermally induced contraction.

### 3.2. Relative Density

The powder mixture composition, sintering parameters and relative density values of the obtained materials are shown in Table 1 and compared to the TiB_2_ sinter produced by SPS at 1700 °C. The relative density of the TiB_2_ sinter is low in comparison to those in References [4,12]. This sinter was produced using the same TiB_2_ powder and in the same sintering conditions as the composites and was used as the referential sample. In general, the relative density of the TiB_2_/Ti_5_Si_3_ composites is increased in comparison to the relative density of the TiB_2_ sinter and increases with the sintering temperature and titanium silicide content. This enhanced density of the produced composites comes at the expense of their hardness. As the applied temperatures of sintering are lower than the melting point of Ti_5_Si_3_, the process does not involve a liquid phase, as is the case of composites with the addition of TiSi_2_ sintered at temperatures above 1500 °C; therefore, such high densities are not obtained. However, our composites can operate at temperatures higher than 1500 °C.

### 3.3. Microstructure 

**Figure 3:**
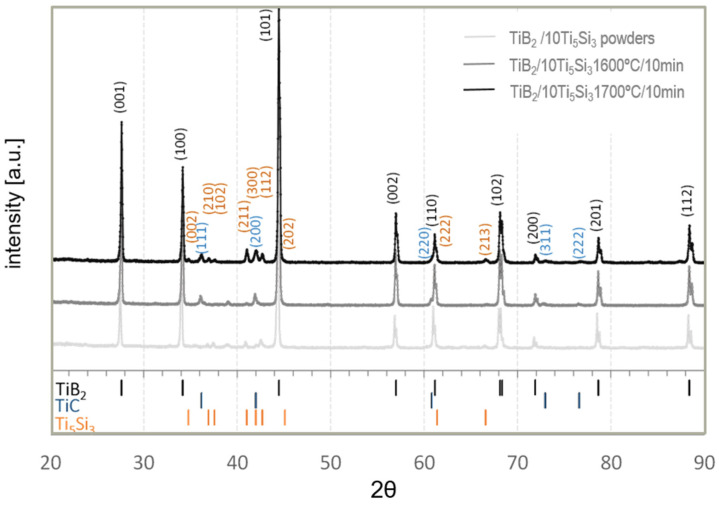
XRD patterns, registered in BB geometry of the TiB_2_ + 10 wt % Ti_5_Si_3_ powder mixture after homogenization, and the TiB_2_/10Ti_5_Si_3_ composites (1600 °C and 1700 °C) with indexed peak positions for the identified phases. ICDD (ref. code) used for the identification of TiB_2_ (01-075-1045), Ti_5_Si_3_ (01-078-1429) and TiC (03-065-8805).

Figure 3 shows the X-ray diffraction spectra registered for the TiB_2_/10 Ti_5_Si_3_ powder mixture after three-step homogenization and the TiB_2_/10 wt % Ti_5_Si_3_ composites. The XRD analysis results confirm that the major phase present in the powder mixture after homogenization and in the produced composites is TiB_2_ (hexagonal closest packed (hcp)). 

The second phase identified in both powder mixtures and the TiB_2_/10 Ti_5_Si_3_ and TiB_2_/20 Ti_5_Si_3_ composites was Ti_5_Si_3_ (D88 crystal structure of the Mn_5_Si_3_). The lattice constants for this silicide identified in the powder sample were a = 7.465 Å and c = 5.168 Å, while in the composites, a = 7.4224–7.4494 Å and c = 5.1501–5.1506 Å were slightly reduced, probably as a result of the applied pressure during sintering. The presence of boron or carbon solid solutions in this silicide that could be formed (Nowotny phase) [13] was not found, and changes in the size of the unit cell parameters did not indicate this. 

Traces of the TiC phase were detected in the composites as a result of the chemical reaction of titanium from the powder mixture and carbon from the graphite components of the sintering die. The TiC content was 2–4 wt % of the produced composites. Although a higher sintering temperature favors an increase in the diffusion rate, no significant differences were observed in the amount of this carbide in our composites. Because the titanium silicide content calculated in the composites is lower than assumed in the initial powder mixtures, with almost the preserved amount of titanium diboride, the formation of titanium carbide probably takes place at the expense of the loss of the titanium silicide. However, other phases forming the Ti-Si or Ti-Si-B system were not identified, which may be due to the evaporation of Si (T_m_ = 1414 °C) or the low content of these phases in the produced composites, below XRD’s detectability. 

**Figure 4:**
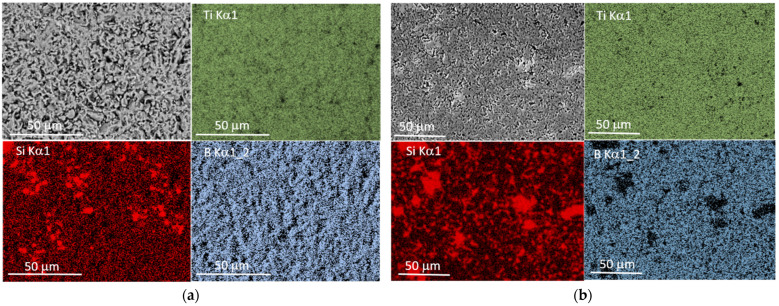
SEM images of the microstructure of the TiB_2_/10 Ti_5_Si_3_ (**a**) and TiB_2_/20Ti_5_Si_3_ (**b**) composites produced by the SPS method (general view with EDS maps showing the Si, B and Ti distribution).

**Figure 5:**
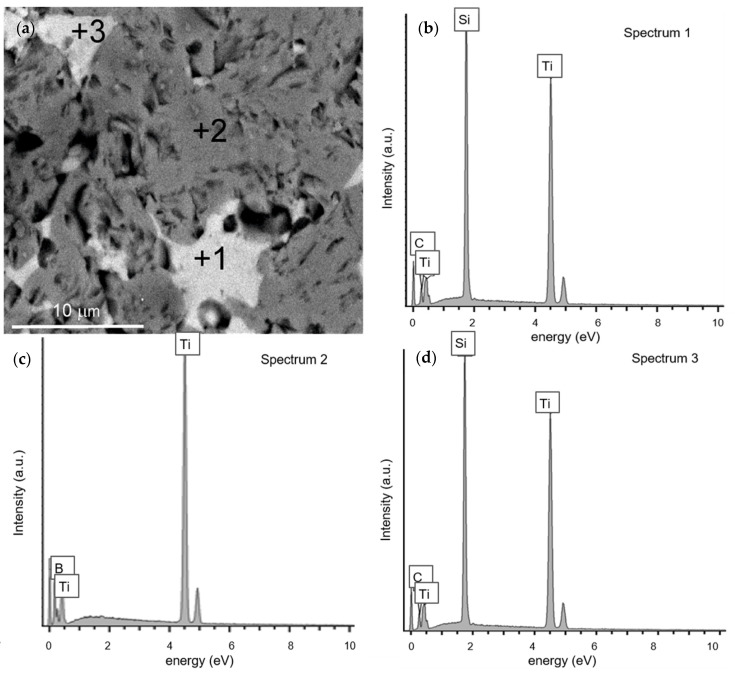
(**a**) SEM image of the microstructure of the TiB_2_10Ti_5_Si_3_ composite (1600 °C/10 min) with marked areas for EDS analysis; (**b**–**d**) EDS spectra taken from them.

SEM observations of the microstructure of the produced composites indicate the presence of two kinds of grains, different in chemical composition (Figure 4 and Figure 5). Figure 4 shows a general SEM image of the microstructure of the composites TiB_2_/10 Ti_5_Si_3_ (Figure 4a) and TiB_2_/20 Ti_5_Si_3_ (Figure 4b) with EDS maps of the elemental distribution of Ti, B and Si. Areas clearly enriched in Si are unevenly dispersed in a homogeneous matrix in terms of the content of B and Ti (Figure 4, EDS maps). The surface of Si-enriched areas increases with the addition of titanium silicide. Some grain growth is observed with sintering temperature. In the SEM images of the microstructure of composites produced with a higher titanium silicide content, Si-rich areas are larger and easily recognizable as light-gray components of the microstructure of an irregular shape distributed in the dark-gray matrix. The darkest areas are pores. Figure 5a shows an SEM image showing the microstructure of TiB_2_/10 Ti_5_Si_3_ with marked areas for EDS analysis and EDS spectra taken from them (Figure 5b–d). In this magnified image, some pores are evident. They most often occur within the grain boundaries, often in the vicinity of grains rich in Si, which may be a result of the evaporation of this element as the sintering temperature was ~150–250 °C above its melting point. Silicon evaporation may be the reason for the failure in achieving the full density of our composites. The loss of this element may cause differences in the Ti/Si ratio in the selected areas of the produced composites, as shown in Figure 5 (EDS Spectra 1 and 3)

### 3.4. Hardness and Friction–Wear Properties

The average hardness values of the produced TiB_2_/Ti_5_Si_3_ composites are given in Table 1 and compared to the hardness measured for the TiB_2_ sinter produced by SPS at 1700 °C/10 min. SPS sintering of TiB_2_ powders at 1600 °C was not effective, and the material densified at the lower sintering temperature was not qualified for further research. 

**Figure 6:**
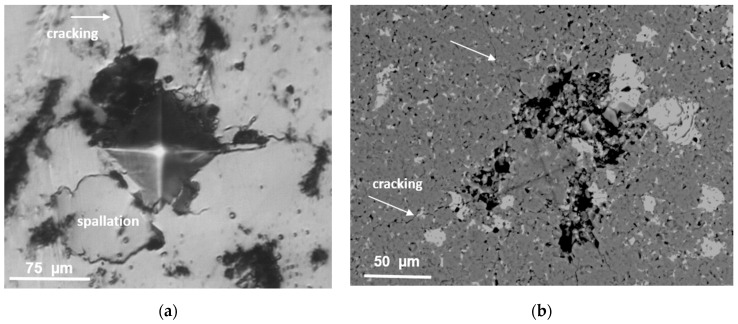
(**a**) Light microscopy image of the surface of the indented area: TiB_2_/10 Ti_5_Si_3_ composite sintered at 1600 °C (**b**); SEM image of the surface of the indented area of the TiB_2_/20 Ti_5_Si_3_ composite sintered at 1600 °C.

Detailed SEM observation of the surfaces of the TiB_2_/Ti_5_Si_3_ composites after the hardness test (Figure 6a,b) indicated that the produced materials were brittle. In the indented area and in its close proximity, there were splinters and/or chippings. Cracking in the corners of the produced indents was evident and propagated intergranular. The susceptibility to chipping and radial cracking formation was increased in the composites containing 20 wt % of titanium silicide addition in comparison to those containing 10 wt % of silicide addition. Strong chipping of the surface under the indenter made the hardness measurement difficult or even impossible. The presence of chipping and spallation of the surface of the specimens near the imprints made it impossible to calculate K_IC_. In general, there are no data that can be found in the field of the microstructure and properties of TiB_2_ composites prepared by the SPS method with Ti_5_Si_3_ as a sintering aid; however, this phase is often identified in composites produced by the sintering of TiB_2_ powder with TiSi_2_ [9,14] or MoSi_2_ [15] sintering additives. The Ti_5_Si_3_ phase occurs in these composites as an unwanted product of the reactions between TiB_2_ and TiSi_2_, occurring during spark plasma sintering at 1400–1650 °C for 10 min but also in composites sintered at a relatively low temperature of 1200 °C but for a longer time duration (i.e., 30 min). In a material containing 5 wt % TiSi_2_, the Ti_5_Si_3_ phase was observed near the interfaces in between the TiB_2_ and TiSi_2_ grains as a layer of 50–100 nm in thickness. Unfortunately, despite the fact that this phase was identified by XRD, its content was not estimated, so its influence on the mechanical properties of produced material is not known. In the absence of information, it remains to compare the measured hardness values to the calculated values on the basis of the rule of mixtures. Assuming TiB_2_ hardness of 2400 HV1 (Table 1) and Ti_5_Si_3_ hardness of 986 HV1, according to [16], the calculated hardness values are ~2258 HV1 for TiB_2_/10 wt % Ti_5_Si_3_ composites and 2117 HV1-for TiB_2_/20 wt % Ti_5_Si_3_, which correspond to the measured values (Table 1). The decrease in the hardness of the produced composites with Ti_5_Si_3_ content is not surprising, as similar degradation of the properties of the TiB_2_-based composites is observed with the addition of other phases beyond 5 wt % of similar hardness to that of TiB_2_, such as MoSi_2_ [15].

**Figure 7:**
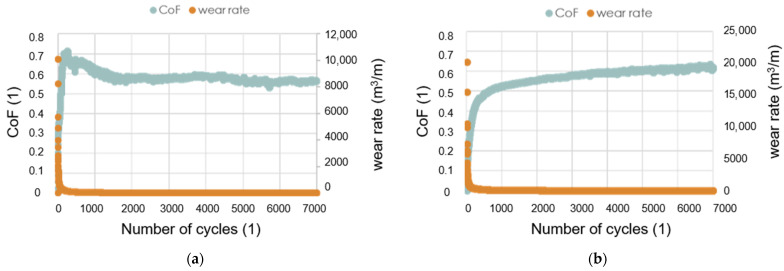
Experimental plots of the CoF and the wear rate of the SPS sinters tested in the dry sliding test with WC as a counterpart: (**a**) TiB_2_/10Ti_5_Si_3_ 1600 °C; (**b**) TiB_2_/20Ti_5_Si_3_ 1700 °C.

**Figure 8:**
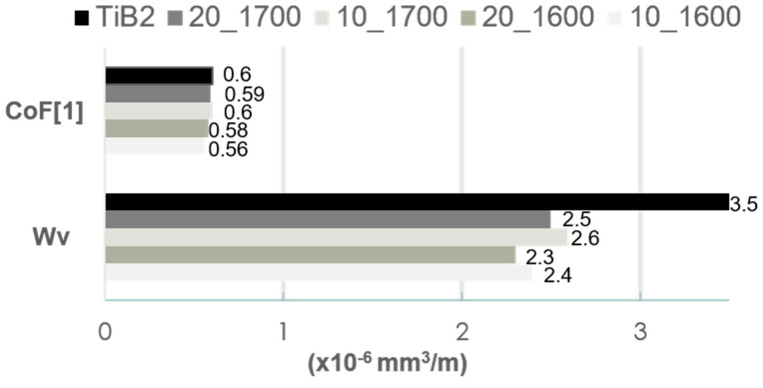
CoF values and volumetric wear indexes (Wv) of the TiB_2_/Ti_5_Si_3_ composites determined in friction contact with WC balls loaded at 5 N.

The friction–wear properties of the TiB_2_/Ti_5_Si_3_ composites were examined in the ball-on-disc test without the use of lubricants. In our experiments, the resistance to wear of the tested samples was determined against a WC ball, as was already been applied in our previous studies for TiB_2_ sinters [17]. Figure 7 shows the CoF and wear rate plots of the composite samples tested in the dry sliding test (ball-on-disc test). The addition of Ti_5_Si_3_ significantly reduces the wear rate of the produced composites. With an increased content of Ti_5_Si_3_, a decrease in the coefficient of friction values is noted at a low number of cycles (N < 2000) but does not influence the average value of the COF, as shown in Figure 8. 

**Figure 9:**
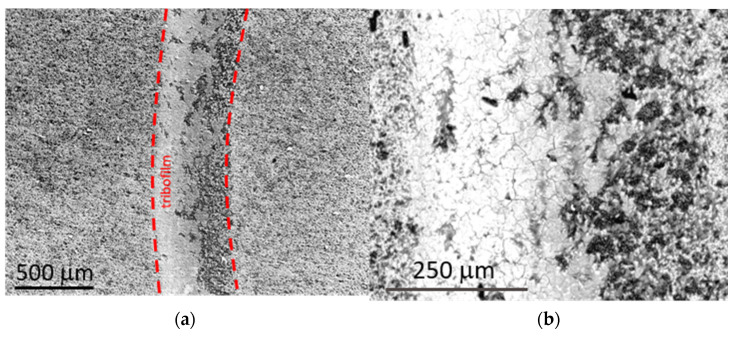
SEM images of the surface of the TiB_2_/20 wt % Ti_5_Si_3_ composite after the ball-on disc test: (**a**) general view (low-vacuum SE image); (**b**) magnified SEM image of the wear track area.

The specific wear rate values Wv are relatively low and similar for all produced composites. In comparison to the value for the referential TiB_2_ sinter tested with the WC ball in the same ball-on-disc test parameters, it was reduced by ~28 %. To understand the cause of the significant increase in the wear resistance with Ti_5_Si_3_ content, the wear tracks were subjected to detailed microstructural observations with the use of a scanning electron microscope (Figure 9) and accompanying analyses of the chemical composition by the EDS method.

**Figure 10:**
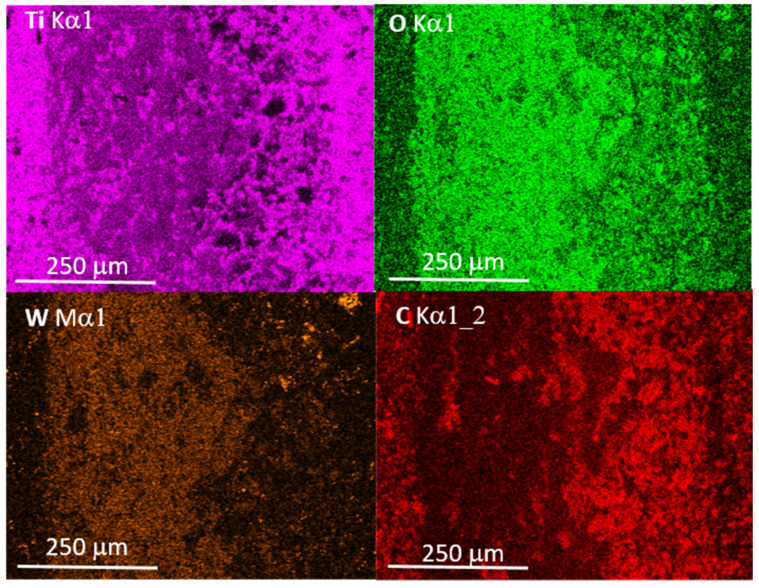
EDS elemental maps of Ti, O, W and C registered from the area presented in Figure 9b.

In the areas of the friction track, numerous grooves were observed, which are typical signs of abrasive wear. EDS maps registered from worn areas indicated the presence of oxygen in the worn area, as well as traces of tungsten and carbon. The presence of boron oxides (and hydroxides), as well as titanium and silicon oxides, was the result of the tribo-oxidation of Ti, B and Si coming from the tested composite samples. Traces of W and C, which were also detected by EDS, come from the WC ball. The distribution of W (Figure 10) also indicates the presence of the tribo-oxidation products of this element. Tungsten and titanium suboxides belong to the group of Magnéli phases with lubricating properties [18,19]. Hydrogenated boron oxides have similar lubricating properties. Their presence in the friction contact area is favorable; however, they do not significantly affect the CoF value (Figure 8). Clearly, tribo-oxidation takes place at the expense of the weight loss of both materials in frictional contact, but the formation of oxides compensates for this loss, which, in turn, has a positive effect on the specific wear rate values.

## 4. Conclusions

The effects of sintering temperature and Ti_5_Si_3_ content on the microstructure of TiB_2_/Ti_5_Si_3_ composites, their density, hardness and friction–wear properties were investigated and discussed. Based on the experimental results and analysis, the following major conclusions were reached:

- Spark plasma sintering is an effective and fast method for the densification of TiB_2_/Ti_5_Si_3_ powders. The addition of 10–20 wt % of Ti_5_Si_3_ is beneficial for the density of TiB_2_/Ti_5_Si_3_ composites. The relative density of the produced composites is up to 98.4% of the theoretical composites.
- SPS sintering of the TiB_2_/Ti_5_Si_3_ initial powders at 1600 °C and 1700 °C results in the formation of TiC due to the effect of carbon diffusion from the graphite components of the sintering die.
- The hardness of the produced composites decreases with Ti_5_Si_3_ content, but their resistance to wear in friction contact with WC increases with it, and for composites containing 20% of this additive, it increases by almost 30%.
- The main mechanism of wear is abrasion. The presence of titanium, tungsten and boron oxides at worn areas indicate the tribo-oxidation reactions of these elements.
- The COF values of TiB_2_/Ti_5_Si_3_ composites in friction contact with WC were in the range of 0.54–0.61, similar to the value of the TiB_2_ sinter.

## Figures and Tables

**Table 1:** Initial phase composition of the powder mixtures and phase composition of the samples after SPS sintering, their hardness and relative density.

Powder Mixture Composition	SPS Conditions Temperature/Time	Phase Compositionafter Sintering	ρ Theoretical (%)	Hardness
TiB_2_/10 wt % Ti_5_Si_3_	1600 °C/35 MPa/10 min	TiB_2_/7 wt % Ti_5_Si_3_/3 wt % TiC	94.8	1961 ± 15 HV1
TiB_2_/10 wt % Ti_5_Si_3_	1700 °C/35 MPa/10 min	TiB_2_/8 wt % Ti_5_Si_3_/4 wt % TiC	96.5	2180 ± 28 HV1
TiB_2_/20 wt % Ti_5_Si_3_	1600 °C/35 MPa/10 min	TiB_2_/16 wt % Ti_5_Si_3_/2 wt % TiC	96.9	1864 ± 40 HV1
TiB_2_/20 wt % Ti_5_Si_3_	1700 °C/35 MPa/10 min	TiB_2_/16 wt % Ti_5_Si_3_/2 wt % TiC	98.2	1953 ± 34 HV1
TiB_2_	1700 °C/35 MPa/10 min	TiB_2_	78.6	2400 ± 45 HV1

## Data Availability

The data presented in this study are available on request from the corresponding author.
